# Supplementary material for: Codominant grasses differ in gene expression under experimental climate extremes in native tallgrass prairie
Source: PeerJ. 2018 Feb 14;6:e4394. doi: 10.7717/peerj.4394 (PMC5816582; doi:10.7717/peerj.4394)
Supplement: Supplemental Information 1 — Supplementary information including supplementary methods not included in the main manuscript, diagram of the experimental heat wave (Fig. S1), effect of the heat wave on canopy temperature and water availability (Fig. S2), microarray normalization plots (Fig. S3), key of samples used in this study (Table S1), and effect of the heat wave and drought on plant physiological characteristics (Fig. S4). [file peerj-06-4394-s001.docx]

**Online Resource for Hoffman, et al. : Codominant grasses differ in gene expression under experimental climate extremes in native tallgrass prairie**

**Supplementary Methods:**

For both the watered and drought plots, a controlled heat wave treatment was achieved by installing pairs of rectangular infrared heating lamps (Kalglo 2000 W, Kalglo Electronic Co Inc., Bethlehem, PA, USA) spaced 1 m apart at the center of each 3 x 6 m plot (Fig. S1). This resulted in a high heat treatment zone (+/- 50 cm of the center of each sampling plot) with a daytime target maximum of +8°C above ambient midday temperature (Fig. S2), and ambient temperature treatment zones (200-300 cm from the mid-point of each 3 x 6 m sampling plot).

Canopy temperatures were measured seven times between 11:00 and 15:00 CDT on June 8^th^ at a distance of +/- 50 cm and 250-300 cm from the center of the sampling plot. These values were averaged over the course of the day to estimate the average canopy temperature difference between ambient and heat wave treatments. Soil moisture was monitored at the center and 250 cm on either side of the center of each sampling plot at a depth of 0-15 cm with 30-cm time-domain reflectometery probes (Model CS616, Campbell Scientific, Logan, Utah, USA) inserted at a 45° angle. On day 9 of the heat wave, sensor data were recorded every 30 min on a CR10X datalogger (Campbell Scientific) and averaged to determine volumetric soil water content for four treatments.

**
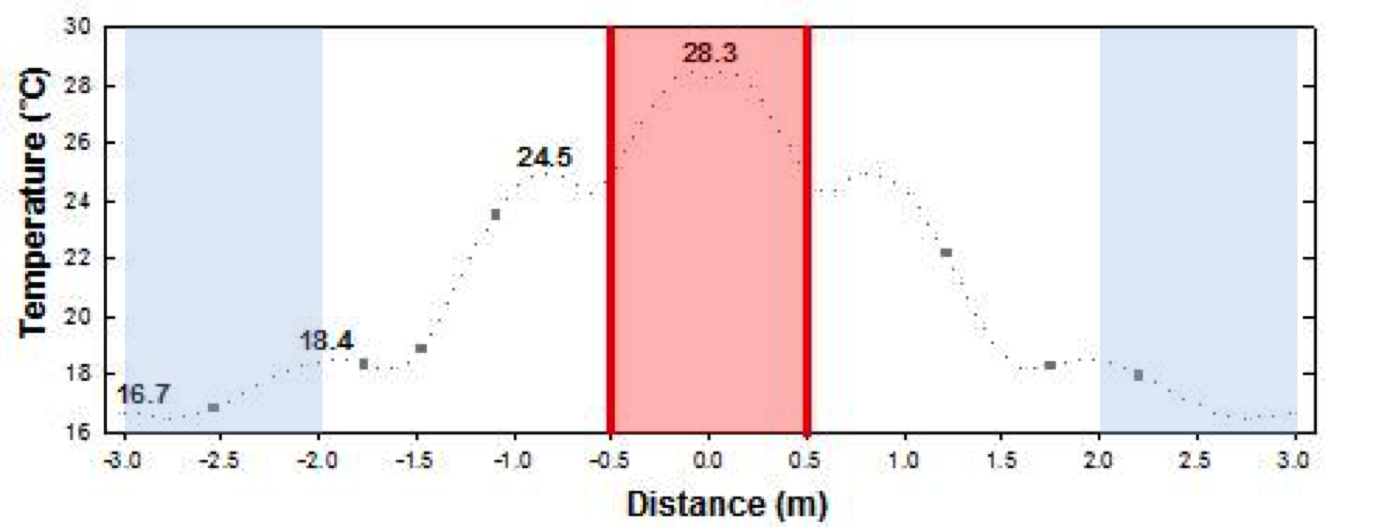
** **Online Resource Figure 1.** The experimental heat wave was imposed by spacing two heaters (red lines) a meter-apart from the center of each 3 x 6 m sampling plot. Plants in the high heat treatment were sampled in the 1 x 3 m zone between these two heaters (red shading), while plants in the ambient treatment were sampled in 1 x 3 m zones located 2 m from the center of the sampling plot (light blue shading). Numbers are mean canopy temperature data at different distances from the center of each sampling plot based on measurements made in early June (see text for details).

**Online Resource Figure S2.** (a) Effect of the heat wave treatment (high heat) on average canopy temperature (b) Effect of the drought and the high heat treatment on mean volumetric soil water content. See text for details.

**Online Resource Figure S3.** Microarray data points, where the x-axis represents the log_10_ intensity of the microarray signal. The y-axis represents the log_2_ expression ratio *A. gerardii* / *S. nutans* (A,B), watered / droughted (C,D), ambient / heated (E,F), and day 4 / day 18 (G,H). Plots on the left-hand side (A,C,E,G) represent non-normalized data, and plots on the right-hand side (B,D,F,H) represent loess normalized data.

**Online Resource Table 1.** Pairing and dye-swap design for the microarray hybridizations. Cy5 dye corresponds to 635 nm wavelength and Cy3 dye corresponds to 532 nm wavelength.

|  | Array Number | Sample Code | Dye | Plot | Temperature treatment |
| --- | --- | --- | --- | --- | --- |
| *Andropogon gerardii* | 13464770 | 12-1-A-4 | Cy3 | 12 | Ambient |
|  |  | 12-7-A-4 | Cy5 | 12 | Heated |
|  | 13464771 | 12-6-A-4 | Cy3 | 12 | Heated |
|  |  | 12-12-A-4 | Cy5 | 12 | Ambient |
|  | 13464772 | 13-1-A-4 | Cy3 | 13 | Ambient |
|  |  | 13-7-A-4 | Cy5 | 13 | Heated |
|  | 13464773 | 13-6-A-4 | Cy3 | 13 | Heated |
|  |  | 13-12-A-4 | Cy5 | 13 | Ambient |
|  | 13464774 | 12-1-A-18 | Cy3 | 12 | Ambient |
|  |  | 12-7-A-18 | Cy5 | 12 | Heated |
|  | 13469185 | 12-6-A-18 | Cy3 | 12 | Heated |
|  |  | 12-12-A-18 | Cy5 | 12 | Ambient |
|  | 13469182 | 13-1-A-18 | Cy3 | 13 | Ambient |
|  |  | 13-7-A-18 | Cy5 | 13 | Heated |
|  | 13469183 | 13-6-A-18 | Cy3 | 13 | Heated |
|  |  | 13-12-A-18 | Cy5 | 13 | Ambient |
| *Sorghastrum nutans* | 13469184 | 12-1-S-4 | Cy3 | 12 | Ambient |
|  |  | 12-7-S-4 | Cy5 | 12 | Heated |
|  | 13469186 | 12-6-S-4 | Cy3 | 12 | Heated |
|  |  | 12-12-S-4 | Cy5 | 12 | Ambient |
|  | 13512650 | 13-1-S-4 | Cy3 | 13 | Ambient |
|  |  | 13-7-S-4 | Cy5 | 13 | Heated |
|  | 13512651 | 13-6-S-4 | Cy3 | 13 | Heated |
|  |  | 13-12-S-4 | Cy5 | 13 | Ambient |
|  | 13512652 | 12-1-S-18 | Cy3 | 12 | Ambient |
|  |  | 12-6-S-18 | Cy5 | 12 | Heated |
|  | 13512653 | 12-6-S-18 | Cy3 | 12 | Heated |
|  |  | 12-12-S-18 | Cy5 | 12 | Ambient |
|  | 13464433 | 13-1-S-18 | Cy3 | 13 | Ambient |
|  |  | 13-7-S-18 | Cy5 | 13 | Heated |
|  | 13464434 | 13-6-S-18 | Cy3 | 13 | Heated |
|  |  | 13-12-S-18 | Cy5 | 13 | Ambient |

**Online Resource Figure 4.** Effect of the heat wave (high heat) treatment on (top) leaf temperature (T_leaf_) and (bottom) leaf water potential (Ψ_mid_) of (left) *Andropogon gerardii* and (right) *Sorghastrum nutans* at day 4 and 18 of the heat wave under watered (W) and drought (D) conditions. T_leaf_ and Ψ_mid_ were significantly different between the high heat and ambient temperature treatments for both species for all four combinations of watered/drought and day of heat wave based on ANOVA (p < 0.05, see text).
